# Effects of Ashwagandha Supplements on Cortisol, Stress, and Anxiety Levels in Adults: A Systematic Review and Meta-Analysis

**DOI:** 10.1192/bjo.2025.10136

**Published:** 2025-06-20

**Authors:** George Bachour, Abdullah Samir, Saddik Haddad, Mohamed Amine Houssaini, Menatallah El Radad

**Affiliations:** ^1^Faculty of Medicine, Tishreen University, Latakia, Syrian Arab Republic; ^2^Cancer Research Centre, Tishreen University Hospital, Latakia, Syrian Arab Republic; ^3^Faculty of Medicine, Ain Shams University, Giza, Egypt; ^4^Faculty of Medicine, Mohammed First University, Oujda, Morocco; ^5^Faculty of Medicine, Misr University for Science and Technology, Cairo, Egypt

## Abstract

**Aims:** *Withania somnifera* widely known as “Ashwagandha”, is a key treatment herb in the Indian system of medicine that has been used for thousands of years. It has anti-inflammatory, neuroprotective, adaptogenic, and immunomodulatory activities.

**Methods:** From inception through September 8, 2024, we searched for randomized clinical trials investigating the effect of ashwagandha on adults. The literature search involved PubMed, Web of Science, Scopus, and Cochrane databases. We included all randomized controlled trials that met the following criteria: (1) conducted on adult participants with stress and/or anxiety; (2) investigated the effects of ashwagandha supplementation compared with a placebo or other active treatments; (3) reported outcomes using validated measures of stress and anxiety. Two reviewers independently extracted the relevant data from the included studies using an Excel sheet. The analysis was carried out using R (version 4.4.1) and the metafor package (version 4.6.0).

**Results:** A total of 15 studies were included with a combined sample size of 873 patients. We found that ashwagandha supplementation significantly reduced anxiety compared with placebo according to the Hamilton Anxiety Rating Scale (HAM-A) before μ =−1.55 (95% CI: −2.45 to −0.65, p=0.0007) and at 8 weeks of treatment μ=−3.52 (95% CI: −6.00 to −1.04, p=0.0053). It has also showed a signifacnt effect in reducing both stress (Perceived Stress Scale (PSS) μ=−4.88 (95% CI: −7.84 to −1.91, p=0.0013)) and cortisol levels μ=−2.3626 (95% CI: −3.2622 to −1.4629), p<0.0001) at 8 weeks of treatment. On the other hand, no improvement in the quality of life has been observed μ =−1.7618 (95% CI: −5.6118 to 2.0881, p=0.3698).

**Conclusion:** Ashwagandha supplementation is safe and effective in reducing stress and anxiety in adult patients. In our study, it resulted in a statistically significant reduction of cortisol levels, PSS scale, and HAM-A scale. However, it showed no statistically significant improvement in the quality of life of participants receiving it.

